# Efficient Labeling of Vesicles with Lipophilic Fluorescent Dyes via the Salt-Change Method

**DOI:** 10.1021/acs.analchem.2c05166

**Published:** 2023-03-29

**Authors:** Minkwon Cha, Sang Hyeok Jeong, Seoyoon Bae, Jun Hyuk Park, Yoonjin Baeg, Dong Woo Han, Sang Soo Kim, Jaehyeon Shin, Jeong Eun Park, Seung Wook Oh, Yong Song Gho, Min Ju Shon

**Affiliations:** † Department of Physics, Pohang University of Science and Technology (POSTECH), Pohang 37673, Republic of Korea; ‡ POSTECH Biotech Center, Pohang University of Science and Technology (POSTECH), Pohang 37673, Republic Korea; § Department of Life Sciences, Pohang University of Science and Technology (POSTECH), Pohang 37673, Republic of Korea; ∥ Biodrone Research Institute, MDimune Inc., Seoul 04790, Republic of Korea; ⊥ School of Interdisciplinary Bioscience and Bioengineering, Pohang University of Science and Technology (POSTECH), Pohang 37673, Republic of Korea

## Abstract

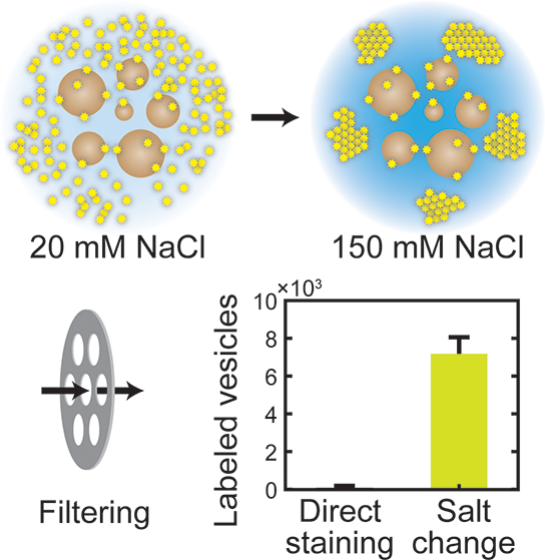

Fluorescent labeling allows for imaging and tracking of vesicles down to single-particle level. Among several options to introduce fluorescence, staining of lipid membranes with lipophilic dyes provides a straightforward approach without interfering with vesicle content. However, incorporating lipophilic molecules into vesicle membranes in an aqueous solution is generally not efficient because of their low water solubility. Here, we describe a simple, fast (<30 min), and highly effective procedure for fluorescent labeling of vesicles including natural extracellular vesicles. By adjusting the ionic strength of the staining buffer with NaCl, the aggregation status of DiI, a representative lipophilic tracer, can be controlled reversibly. Using cell-derived vesicles as a model system, we show that dispersion of DiI under low-salt condition improved its incorporation into vesicles by a factor of 290. In addition, increasing NaCl concentration after labeling induced free dye molecules to form aggregates, which can be filtered and thus effectively removed without ultracentrifugation. We consistently observed 6- to 85-fold increases in the labeled vesicle count across different types of dyes and vesicles. The method is expected to reduce the concern about off-target labeling resulting from the use of high concentrations of dyes.

## Introduction

Fluorescent labeling of extracellular vesicles (EVs) offers a unique approach to study physical and functional properties of vesicles. For example, tracking diffusion of EVs under fluorescence microscopes can measure the size distribution of EV population, similarly to nanoparticle tracking analysis by light scattering.^1,2^ More sophisticated techniques also exist for super-resolution imaging, multiplexed measurements, or flow cytometry of EVs.^3,4^ Lastly, docking of EVs on live cell membranes and their subsequent uptake can be followed in a quantitative manner.^5−7^ Regardless of the specific properties under investigation, the results can be often confounding due to the underlying heterogeneity in vesicles, in which case the analysis must be conducted at the single-vesicle level to faithfully reconstruct the ensemble properties.^8^ Therefore, effective and unbiased, homogeneous labeling with bright fluorescent dyes that visualizes individual vesicles is a prerequisite for EV analysis by fluorescence.

Several methods to fluorescently label EVs are available: immunostaining of surface proteins, internal protein tagging with membrane-permeable dyes, use of water-soluble dyes inside vesicles, or membrane staining with lipophilic dyes.^9^ Unfortunately, vesicle labeling via proteins would be biased by the abundance of proteins and potentially interfere with the following functional characterization dependent on the targeted proteins. Water-soluble dyes behave largely independent of the vesicle content, but cannot be internalized into preformed vesicles such as purified EVs due to the membrane barrier. Although some nonfluorescent, membrane-permeant molecules, such as carboxyfluorescein diacetate succinimidyl ester (CFDA-SE), can passively diffuse into vesicles and then become fluorescent,^3^ they only work with vesicles containing active esterases and therefore will be biased by the vesicle content. Membrane staining with lipophilic tracers offers unbiased and bright labeling: a variety of cyanine-derivative dyes with single-molecule sensitivity are developed across the entire spectrum of visible light.^10^ Since a typical, 100 nm vesicle carries ∼80000 lipid molecules, introducing only 0.01 mol % of lipophilic dyes will yield ∼8 dye molecules per vesicle on average, sufficient for single-particle tracking. Additionally, these molecules naturally exhibit a large increase in fluorescence upon partitioning into the membrane, further contributing to the high signal-to-noise ratio against free dye molecules.

Notably, however, vesicle staining with lipophilic dyes often suffers from confounding factors, such as the complications from off-target labeling of lipoproteins and free-dye aggregates that are similar in size to EVs.^11−13^ These problems are associated with the use of high concentrations of dyes that are used to force the incorporation of lipophilic molecules into closed, preformed vesicles. Although slightly soluble in water, most of the lipophilic molecules are kinetically trapped in large aggregates, so the passage from aggregates to membranes is unfavorable. One can drive the equilibrium toward vesicles by increasing dye concentrations, but at the expense of additional sample processing to remove the large amount of free dyes, which is frequently accompanied by severe loss of vesicles. Moreover, a recent work suggests that the incorporation of large dye aggregates may also lead to a significant increase in vesicle size.^14^ Consequently, an effective method to fluorescently label vesicles with high sensitivity, high specificity, and simplicity to preserve the quality and quantity of vesicle samples is desirable.

**Figure 1:**
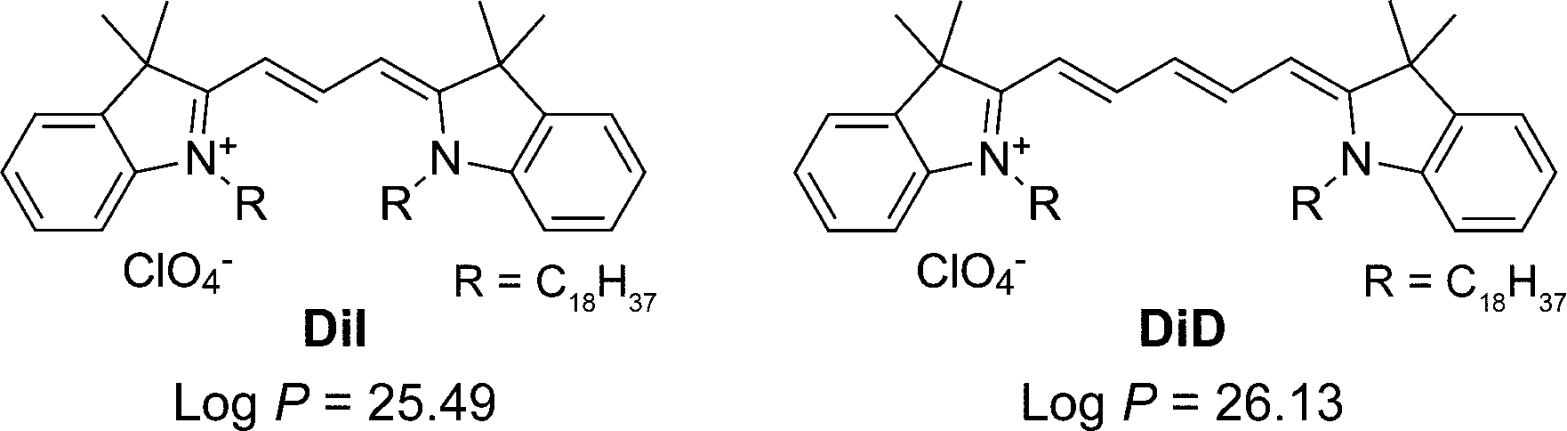
Chemical structures of lipophilic fluorescent probes for vesicles. Lipophilicity (log *P*) values for octanol/water partition coefficient were calculated using a web tool (SwissADME^15^) with a XLOGP3 model.^23^

Here, we describe an improved method for the fluorescent labeling of vesicle samples, including EVs. This method is simple, fast, and effective both in improving the labeling efficiency and in removing unwanted free dyes. By characterizing the aggregation status of a lipophilic tracer DiI (Figure 1) as a function of NaCl concentration using total internal reflection fluorescence (TIRF) microscopy, we observed that ∼150 mM NaCl, a typical constituent of standard phosphate-buffered saline (PBS), induces a pronounced aggregation of DiI molecules that potentially inhibit their incorporation into vesicle membranes. The aggregation was reversible, as shown by the dispersion of aggregates at a lower concentration of NaCl. Therefore, to improve labeling efficiency, we performed labeling and free-dye removal in separate steps with distinct NaCl concentrations. The free-dye aggregates, due to their large size, were completely removed by simple filtering without requiring ultracentrifugation, along with minimal vesicle loss. The resulting efficiency of labeling was consistently improved across multiple types of lipophilic dyes and vesicle samples by at least an order of magnitude compared with a standard labeling procedure. The increased efficiency in turn allows labeling with lower concentrations of dyes than usual, and therefore reduces the concern about free-dye aggregates that are preferentially formed at high concentrations. The wide applicability of the proposed method will likely facilitate the use of fluorescently labeled vesicles in many different types of EV analysis.

## Materials and Methods

### Fluorescent Labeling of Vesicles

Preparation of vesicles are described in the Supporting Information. For the salt-change labeling, vesicle samples in PBS were diluted to 10^10^ particles/mL with ultrapure water to lower NaCl concentration to below 20 mM and reacted with 2 μM DiI or DiD for 20 min at 37 °C. A small volume of PBS concentrate (10×) was then added to increase NaCl concentration to ∼150 mM. For direct staining, the same concentration of dyes was directly applied to vesicles in regular PBS with 150 mM NaCl. The mixture was filtered through a 0.2 μm syringe filter (Advantec, 03CP020AS) to remove aggregates of dye molecules and subjected to further experiments. Labeling results were verified by imaging of either free-floating or anti-CD63-bound vesicles on a TIRF microscope (see Video S1 for the representative results) as described in the Supporting Information.

## Results

### NaCl Dependence of DiI Aggregation

Although lipophilic dyes are commercially available as powders or as solutions in organic solvents such as ethanol, dimethylformamide (DMF), or dimethyl sulfoxide (DMSO), they need to be transferred to aqueous solutions for vesicle labeling not to disrupt vesicles and the embedded proteins. This requirement poses a challenge because the water solubility of lipophilic molecules is generally low, as shown by their high lipophilicity (calculated log *P* values for the octanol/water partition coefficient^15^ are given in Figure 1). Therefore, we first directly examined DiI molecules dissolved in aqueous solutions by single-molecule TIRF microscopy^16^ (Figure S1A). After introducing DiI solutions into a glass flow cell, the fluorescent particles floating by near the glass surface were illuminated.

We first imaged 2 μM DiI solution in a buffer with ∼150 mM NaCl, a physiological and typical condition for common buffers including PBS. A small number of bright, slowly moving particles were detected (Figure S1A), which are likely large aggregates of DiI rather than single molecules. Since most fluorescent dyes including DiI exhibits aggregation-caused quenching of fluorescence intensity, the brightness of the particles would actually underestimate the number of dye molecules per particle. Indeed, these aggregates were completely removed by filtering the solution through 0.2 μm pores (Figure S1A,B), implying that they are mostly micron-sized. These large aggregates are likely inefficient at labeling vesicular membranes and may cause an increase in vesicle size after labeling.^14^

We therefore attempted to improve the solubilization of DiI by decreasing NaCl concentration. The aggregates gradually dispersed as NaCl concentration was lowered to ∼20 mM, as shown by the increase of relatively dim particles (Figure S1A–C). Importantly, we checked that these changes to particles occurred while the total amount of dye molecules and their fluorescence remained constant: After solubilizing the dye aggregates completely with detergent (0.1% Triton X-100), the overall fluorescence intensity from the solution was measured to be the same across the concentrations of NaCl we tested (Figure S1D). In contrast, the fluorescence from the buffer with 155 mM NaCl almost completely disappeared after micropore filtering (Figure S1D; ∼5 nM DiI left from the original 2 μM solution), suggesting that most of the dye molecules in this condition were trapped in the aggregates and subsequently removed.

### Improvement of Fluorescent Labeling by the Salt-Change Method

**Figure 2:**
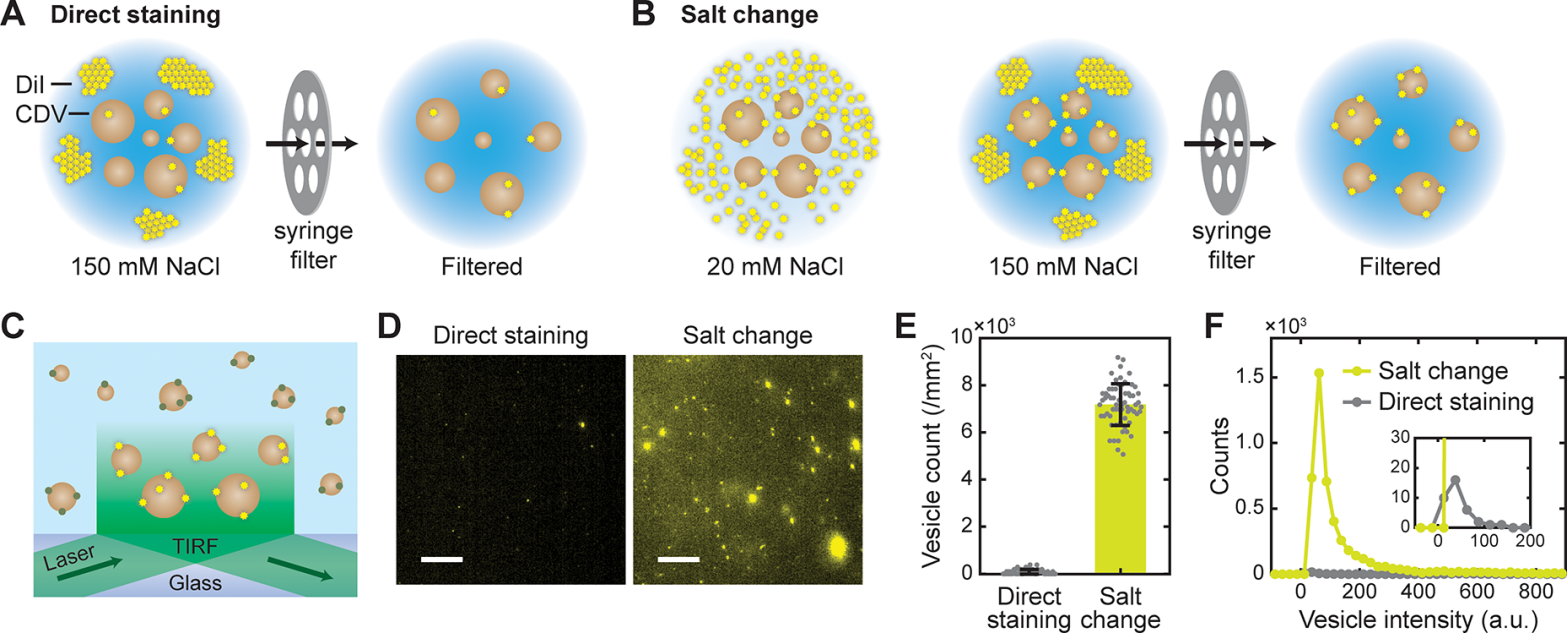
Fluorescent labeling of vesicles by salt-change method. (A, B) Schematics of direct staining (A) and salt-change (B) method for the labeling of cell-derived vesicles (CDVs) with DiI in an aqueous buffer. In the salt-change method, staining was performed in a low-salt buffer, then [NaCl] was increased to 150 mM to promote aggregation of DiI, and the aggregates were removed by filtration. (C) Schematic of single-vesicle imaging of DiI-labeled CDVs by TIRF microscopy. (D) Representative fluorescent images of DiI-labeled vesicles. Scale, 10 μm. (E) Numbers of DiI-labeled vesicles obtained by direct staining and salt-change method observed in TIRF images. Error bars, mean ± s.d. of *n* = 63 images. (F) Distributions of fluorescence intensity for the labeled vesicles. Inset, close-up view of the same curves; *n* = 56 (direct staining), *n* = 4671 (salt change) spots.

The above results suggest that dispersion of DiI in a buffer with a low concentration of NaCl can potentiate membrane partitioning of DiI and that the excess dye can be removed by inducing its aggregation at a higher concentration of NaCl. We therefore exploited this reversible aggregation of DiI to improve the labeling of vesicles (Figure 2). To verify the general applicability of labeling procedures, we employed cell-derived vesicles (CDVs) as model EVs that are similar in size to large exosomes and small microvesicles.^17^ CDVs from human natural killer cells (hereafter called NK-CDVs) were prepared by using a published procedure,^18^ and labeled them with DiI (1,1′-dioctadecyl-3,3,3′,3′-tetramethylindocarbocyanine; DiIC_18_(3)), a lipophilic fluorescent tracer for labeling lipid membranes.

For the side-by-side comparison of labeling efficiency, two labeling methods were applied to NK-CDVs: (a) “Direct staining” performed with 150 mM NaCl, that is, in regular PBS (Figure 2A), and (b) “Salt-change” approach in which the labeling with and removal of DiI were performed separately in buffers with distinct ionic strength (Figure 2B). In the latter method, after staining NK-CDVs in a low-salt buffer ([NaCl] < 20 mM), we raised NaCl concentration to ∼150 mM to induce aggregation of free dye molecules, then filtered the solution using a regular syringe filter. This filtering step was applied also to the direct staining procedure for a fair comparison of labeling results and vesicle yield. The labeled CDVs were then visualized using a TIRF microscope^16^ (Figure 2C).

CDVs after direct staining displayed only a small number of dim particles (Figure 2D; see also Video S1). Since the CDVs were prepared at a fairly high concentration (∼10^10^ particles/mL), we expected much more particles to be present in the field of view, and therefore, it was very unlikely that all CDVs were successfully labeled by the direct staining method. Although the observed level of labeling efficiency might be suitable for bulk assays that probe many vesicles at the same time (e.g., cellular uptake of vesicles), the labeled CDVs were neither sufficiently abundant nor sufficiently bright for quantitative measurements at the single-vesicle level. According to our observations of NaCl concentration-dependent DiI aggregation, we argued that the low labeling efficiency would stem from poor solubilization of lipophilic dyes in the staining buffer.^19^ In stark contrast, the salt-change method increased the number of bright fluorescent vesicles 85 ± 10 times (Figure 2D,E; see also Video S1), and their average brightness also increased 2.3 times compared to vesicles stained in PBS with 150 mM NaCl (Figure 2F). The simultaneous increase in number and brightness of fluorescent vesicles implies that the overall DiI incorporation (estimated from the areas under the curves in Figure 2F) was improved by a factor of 290.

To accurately measure the labeling density (i.e., number of DiI molecules per vesicle), the labeled NK-CDVs were stably captured on a surface and their fluorescence intensity was measured (Figure S2A; see Supporting Information for the method). We estimated the number of DiI molecules in each vesicle from the ratio of the initial fluorescence to photobleaching step size (Figure S2B–D). Each CDV typically carried 1–3 molecules of DiI, and these numbers followed a Poisson distribution as expected (Figure S2E). The results imply that only a small fraction of the CDVs remained unlabeled and, at the same time, that the mole fraction of DiI in vesicle membranes was <10^–4^ (less than 10 dye molecules vs ∼10^5^ lipid molecules; see Supporting Information for the full calculation). Therefore, the labeling density we achieved was sufficient for single-vesicle imaging, but unlikely to disrupt the native properties of the membrane.

### Applications to Other Vesicles and Dyes

To test whether the salt-change labeling method can be applied to other vesicles, we first prepared another sample of CDVs from umbilical cord mesenchymal stem cells (UCMSC-CDVs) and labeled them with DiI. Again, the salt-change method showed a dramatic improvement in labeling efficiency (Figure S3A,B), consistent with the results for NK-CDVs. It is remarkable that the proposed method was much more effective than adding dimethyl sulfoxide (DMSO) to the staining buffer (Figure S3A,B), a common approach to improve the solubility of lipophilic dyes. Also, if the syringe filters for dye removal were not rinsed with buffer (2 mL of PBS) before use, we noticed that the vesicle yield decreased slightly (by 17%; Figure S3C,D), possibly due to the trace wetting agents in the off-the-shelf cellulose acetate filter membranes.

**Figure 3:**
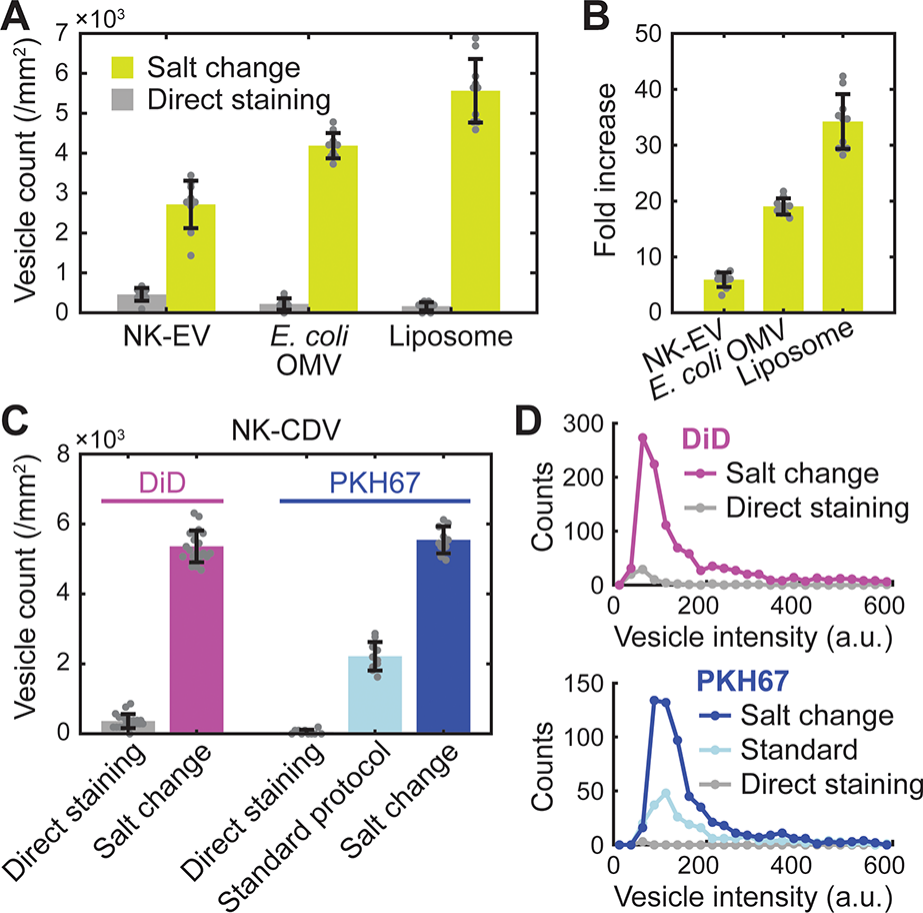
Applications of salt-change labeling. (A) Fluorescent labeling of mammalian EVs from NK-92 cells (NK-EV), bacterial outer-membrane vesicles from *E. coli* W3110 (*E. coli* OMV), and synthetic liposomes with comparison of the labeling methods. (B) Fold increase in labeling efficiency (vesicle count) calculated from (A). (C) Comparison of labeling methods for DiD and PKH67. For PKH67, results from a standard protocol (Supporting Information) is also shown. Error bars, mean ± s.d. of *n* = 20 (DiD) and 10 (PKH67) images. (D) Distributions of fluorescence intensity for the DiD- and PKH67-labeled vesicles shown in (C).

Importantly, the labeling method was also successfully applied to naturally occurring EVs (Figure 3A,B). We prepared two types of EVs: mammalian EVs from human natural killer cells (NK-92; “NK-EV”) and bacterial outer-membrane vesicles (OMVs) from *Escherichia coli* W3110 (a widely used wild-type strain) (“*E. coli* OMV”), following a published procedure.^20,21^ Applying the salt-change labeling to these vesicles, we again obtained a great improvement in labeling efficiency over direct staining in a high-salt buffer (6-fold and 19-fold for NK-EVs and *E. coli* OMVs, respectively; Figure 3A,B). Furthermore, the method also proved to be useful in labeling preformed liposomes consisting purely of synthetic POPC (palmitoyloleoylphosphatidylcholine) lipids (Figure 3A), with the greatest (34-fold) increase in vesicle count (Figure 3B). Since CDVs, mammalian and bacterial EVs, and liposomes fairly differ in lipid and protein composition and size distribution, our results clearly suggest that the proposed salt-change method will be generally applicable to most types of native and synthetic vesicles.

Experiments with two other lipophilic dyes DiD (1,1′-dioctadecyl-3,3,3′,3′-tetramethylindodicarbocyanine, DiIC_18_(5); Figure 1) and PKH67 showed similar results in CDV staining (Figure 3C,D). Strikingly, the salt-change method applied to PKH67 dyes performed better than a recommended standard protocol (from Sigma-Aldrich) that used 3 times more vesicles and 6 times more dyes for comparable results, improving both the number and brightness of the stained vesicles. The same vesicles were barely detected after labeling by direct staining with PKH67. The moderate improvement with the standard protocol over direct staining can be explained by the use of Diluent C, a commercial salt-free isotonic solution supplied for general membrane labeling (from Sigma-Aldrich). Although the exact structure of PKH67 is unpublished, it is reported (in the product description by Sigma-Aldrich) to contain an aliphatic tail longer than PKH2 that has a C_22_ tail. It is therefore expected to be highly lipophilic, and presumably, the labeling strategy proved successful similarly to DiI and DiD. Together, these results demonstrate the broad applicability of the salt-change method for the fluorescent labeling of preformed vesicles with lipophilic dyes.

### Integrity and Recovery of Vesicles after Labeling

**Figure 4:**
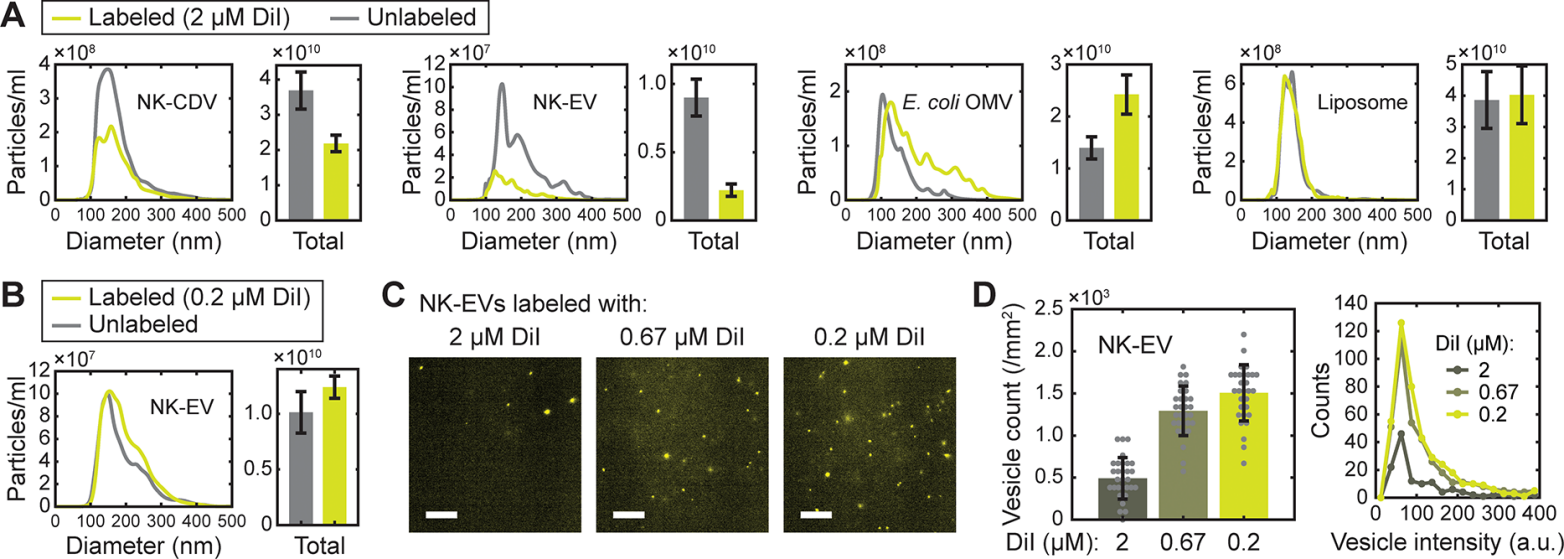
Size distribution and recovery of vesicles after salt-change labeling. (A) Nanoparticle tracking analysis (NTA) of vesicle size distribution for the unlabeled (gray) and 2 μM DiI-labeled (green) vesicles via the salt-change method. The size distributions (left panels) are shown with the corresponding total particle concentrations on right (bars). (B) NTA results for the salt-change labeling of NK-EVs with 0.2 μM DiI. In (A) and (B), error bars represent mean ± s.d. of *n* = 26–29 measurements. (C) Representative images of DiI-labeled EVs prepared by salt-change labeling; scale, 20 μm. (D) Number of DiI-labeled vesicles prepared with the indicated concentrations of DiI. Vesicle counts (left) from images such as shown in (C) are shown with the corresponding intensity distribution (right). Error bars, mean ± s.d. of *n* = 30 images.

Consistent with the small amount of dye molecules per vesicle, the size distribution of NK-CDVs, as measured by nanoparticle tracking analysis (median diameter of ∼150 nm), was not distorted by the salt-change labeling (Figure 4A). Although the nominal pore size (0.2 μm) of the syringe filter for dye removal was close to the size of CDVs, the actual pore sizes in the cellulose acetate filter membrane are heterogeneous and allow the passage of vesicles slightly larger than 0.2 μm, so the vesicles between 200 and 400 nm were not appreciably cut off. Overall, 60% of the vesicles were recovered after salt-change labeling of NK-CDVs (Figure 4A). It is remarkable that free DiI molecules were almost completely removed by the same filtering process (Figure S1D), thus, the size difference between vesicles and free-dye aggregates could be successfully exploited to purify labeled vesicles.

Vesicle sizes were largely maintained for the other types of vesicles (NK-EVs, *E. coli* OMVs, and synthetic liposomes), too, but the yield somewhat depended on vesicle type (Figure 4A). While synthetic liposomes were most reliably recovered with a minimal change in size distribution, NK-EVs and *E. coli* OMVs showed opposite results in the obtained numbers of vesicles (25% and 170%, respectively). The increase in OMV number can be rationalized by the suboptimal detection of small particles in NTA measurements, which became detected upon labeling. Notably, although the recovery of NK-EV was relatively low when 2 μM DiI was used, the yield was completely restored by using 0.2–0.67 μM DiI (Figure 4B). In fact, this condition improved the labeling efficiency as well (Figure 4C,D and Video S2), suggesting that an optimal dye concentration needs to be determined empirically for a given sample of vesicles.

**Figure 5:**
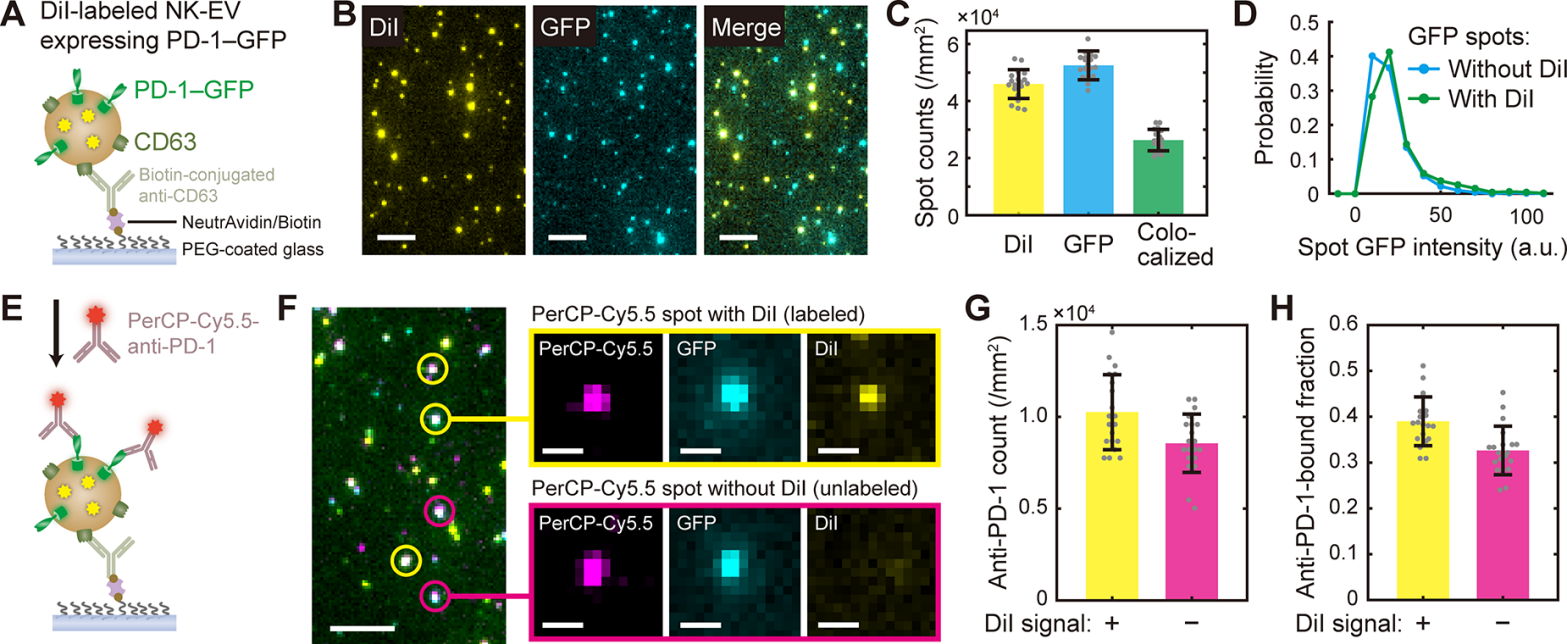
Integrity of vesicle proteins after salt-change labeling (A) Schematic of single-vesicle pull-down and imaging of DiI-labeled NK-EVs containing PD-1-GFP. (B) Representative fluorescence images from the experiments described in (A). Scale, 5 μm. (C) Colocalization of DiI and GFP spots from the NK-EV images such as shown in (B). (D) GFP intensity distribution for the spots with DiI (labeled) and without DiI (unlabeled). (E) Schematic of NK-EV detection with PerCP-Cy5.5-conjugated PD-1 antibody. (F) Representative fluorescence images from the experiments described in (E). Insets show magnified views of the selected spots with and without DiI. Scale, 5 μm (on left) and 1 μm (insets). (G) Numbers of anti-PD-1 (PerCP-Cy5.5) spots with (yellow) and without (magenta) DiI signal. (H) Fraction of GFP spots detected by anti-PD-1 as a function of the presence of DiI.

For downstream uses, retaining the native structures and functions of vesicle proteins after salt-change labeling would be important. To this end, we tested whether the labeled vesicles can be captured by antibodies to a common component of EVs, CD63, and then detected by another antibody toward a cargo protein.^22^ We prepared EVs from NK-92 cells that overexpressed PD-1-GFP, labeled them with DiI by using the salt-change method, and then pulled them down onto a polyethylene glycol (PEG)-coated glass surface with anti-CD63 (Figure 5A). The resulting surface showed bright DiI spots that are colocalized (57%) with GFP spots (Figure 5B,C), indicating successful capturing of NK-EVs via CD63 and therefore the presence of intact CD63 molecules on EV membranes after labeling. By comparing the GFP spots with and without DiI, we noticed that the distribution of GFP intensity was not altered by the presence of DiI (Figure 5D), suggesting that the amount of PD-1-GFP per vesicle was not perturbed (e.g., from leakage or vesicle fusion) during the labeling procedure. Additionally, when a PD-1 antibody was introduced over the captured EVs (Figure 5E), the fluorescence from anti-PD-1 colocalized with GFP spots with a high efficiency (Figure 5F,G), verifying the presence of PD-1. Since the detection efficiency (as measured by anti-PD-1-bound fraction) did not depend on the presence of DiI label (Figure 5H), we conclude that the incorporation of DiI molecules did not change the affinity between PD-1 and its antibody, and suggest that such native interactions can be preserved after salt-change labeling.

## Discussion

Successful labeling of vesicles with bright fluorescent dyes is a prerequisite for quantitative analysis of vesicles via fluorescence. Vesicles are commonly labeled by targeting surface proteins, but this method not only depends on protein composition but also interfere with downstream measurements. The membrane staining procedure introduced here addresses many challenges associated with vesicle labeling: labeling was unbiased and effective for all types of vesicles and dyes we tested because the dyes target generic lipid bilayers (Figures 2 and 3); virtually all free-dye particles were removed by NaCl-induced aggregation and subsequent filtering (Figure S1); and the recovery of input vesicles was satisfactory (Figure 4).

The two-orders-of-magnitude improvement in labeling efficiency with the salt-change method is impressive given its simple steps, and therefore can potentially substitute complex labeling and purification protocols (such as the standard protocol for PKH67 we used for comparison), even for researchers with access to an ultracentrifugation system.^3^ The method also does not involve any proprietary formulation (e.g., diluent C used with PKH dyes^12^) and can be finished within 30 min in a regular wet lab. Improving the labeling efficiency also allows the use of lower concentrations of dyes and vesicles and thereby reduce the possibility of nonspecific labeling. We expect this method to apply to clinically obtained EVs from liquid biopsy, which will particularly benefit from effective labeling because they are usually limited in amount.

One concern with the salt-change labeling is that ionic strength change may induce osmotic stress on vesicles and membrane proteins. We verified that the selected proteins (endogenous CD63 and overexpressed PD-1) were still recognized by antibodies after labeling (Figure 5). Although these were minimal tests, we think the salt-change labeling method does not seriously sacrifice the functionality of vesicles given that the incorporated numbers of dye molecules were small (∼2 on average) and the size and shape of the vesicles were largely maintained. Also, it would be important to be aware of potential contamination from the syringe filters, especially because some filter membranes contain wetting surfactants that can destroy vesicles. We showed that prerinsing of the filter to remove aqueous extractables can gently improve the recovery of vesicles (Figure S3C,D), but the results will likely depend on the specific filter models in use.

## Conclusion

In this study, we investigated fluorescent labeling of vesicles with lipophilic dyes, revealing a critical dependence on NaCl concentration. We exploited the reversible aggregation of DiI molecules both to improve labeling efficiency and to remove free dye molecules from the vesicle solution. The salt-change labeling method was shown to be widely applicable to many types of vesicles and dyes, without noticeably degrading the functional properties of vesicle samples and content proteins. We expect that this protocol will be useful in a broad spectrum of fluorescence-based assays interrogating natural EVs and engineered nanovesicles.

## Supporting Information Available

Supplementary Methods for the preparation of vesicles, NTA measurements, PKH67 labeling, TIRF microscopy and image analysis including labeling efficiency; Supplementary Figures on NaCl dependence of DiI aggregation (Figure S1), estimation of labeling efficiency (Figure S2), and comparison of CDV labeling efficiency (Figure S3) (PDF)
NK-CDVs labeled by direct staining (Video S1) (AVI)
Effect of DiI concentration on salt-change labeling of NK-EVs (Video S2) (AVI)

## Supplementary Material

ac2c05166_si_001.pdf

ac2c05166_si_002.avi

ac2c05166_si_003.avi
