# Supplementary material for: Efficient Labeling of Vesicles with Lipophilic Fluorescent Dyes via the Salt-Change Method
Source: Anal Chem. 2023 Mar 29;95(14):5843–9. doi: 10.1021/acs.analchem.2c05166 (PMC10100391; doi:10.1021/acs.analchem.2c05166)
Supplement: Supplementary file 1 — ac2c05166_si_001.pdf [file ac2c05166_si_001.pdf]

Supporting Information for:

Efficient Labeling of Vesicles with Lipophilic Fluorescent dyes via the Salt-Change Method

Cha *et al.*

This PDF file includes:

Supplementary Methods

Supplementary Figures S1–S3

Supplementary Video Legends

Supplementary References

Supplementary Methods

Preparation of CDVs

NK- and UCMSC-CDVs were prepared as previously described.[1] Briefly, cells were suspended in PBS at a concentration of 10^5–10^6 particles/ml, and then serially extruded through polycarbonate track-etched hydrophilic membrane filters (Cytiva, Whatman) with pore sizes of 5, 1, and 0.4 µm for NK-CDVs, and 10, 3, and 0.4 µm for UCMSC-CDVs. The resulting solutions of CDVs were treated with DNase (Merck Millipore, Benzonase Nuclease HC) to remove DNA outside CDVs, and aggregates were removed by centrifugation at 3,000×g for 10 min. The CDVs were further purified by a tangential flow filtration system (Repligen, KrosFlo KR2i) to eliminate impurities such as free proteins or nucleic acids, followed by final filtration through 0.45-µm pores to remove large, non-vesicular structures. The concentration and size distribution of CDVs were measured using nanoparticle tracking analysis (Particle Metrix, Zetaview). The purified CDVs were stored in PBS at a concentration of $\sim 1\times10^{11}$ particles/ml and kept at −80 °C until use.

Preparation of natural EVs

Mammalian and bacterial EVs for the application of vesicle labeling method were prepared as previously described.[2,3] For mammalian EVs, culture media (α-MEM supplemented with 2 mM L-glutamine, 0.2 mM myo-inositol, 0.02 mM folic acid, 0.1 mM 2-mercaptoethanol, 20 ng/ml recombinant human IL-2, 12.5% FBS, 12.5% horse serum and 1% penicillin/streptomycin) from human NK-92 cells grown for 24 h with 10% EV-depleted FBS were centrifuged once at 500×g and twice at 2,000×g to remove cells. The supernatant was concentrated using a MinimateTM TFF capsule with 100-kDa membrane (Pall Corporation). The concentrated supernatant was placed over 0.8 M and 2.0 M sucrose cushion and centrifuged at 100,000×g for 2 h at 4 °C, and then EVs

were collected from the interface between the sucrose cushion. For bacterial OMVs, cultured lysogeny broth from *E. coli* W3110 cells grown until the OD600 reached 1.5 was centrifuged twice at 6,000×g to remove cells. The supernatant was filtered through 0.45-μm pores and was concentrated with a QuixStandTM benchtop system using 100-kDa hollow fiber membranes (Amersham Biosciences). The concentrated supernatant was filtered through 0.22-μm pores, and then OMVs were pelleted by ultracentrifugation at 150,000×g for 2 h at 4 °C. The EVs and OMVs were further purified through iodixanol buoyant density gradient ultracentrifugation.

Preparation of PD-1–GFP-loaded NK-92 EVs

To construct an NK-92 cell line expressing PD-1–GFP, we purchased a lentivirus vector with cDNAs for murine PDCD1 gene (NM008798) and mGFP (Origene MR227347L4):

PD-1 sequence:
ATGTGGGTCCGGCAGGTACCCTGGTCATTCACTTGGGCTGTGCTGCAGTTGAGCTGGCAATCAGGGTG
GCTTCTAGAGGTCCCCAATGGGCCCTGGAGGTCCCTCACCTTCTACCCAGCCTGGCTCACAGTGTCAGA
GGGAGCAAATGCCACCTTCACCTGCAGCTTGTCCAACTGGTCGGAGGATCTTATGCTGAACTGGAACC
GCCTGAGTCCCAGCAACCAGACTGAAAAACAGGCCGCCTTCTGTAATGGTTTGAGCCAACCCGTCCAG
GATGCCCGCTTCCAGATCATACAGCTGCCCAACAGGCATGACTTCCACATGAACATCCTTGACACACG
GCGCAATGACAGTGGCATCTACCTCTGTGGGGCCATCTCCCTGCACCCCAAGGCAAAAATCGAGGAGA
GCCCTGGAGCAGAGCTCGTGGTAACAGAGAGAATCCTGGAGACCTCAACAAGATATCCCAGCCCCTC
GCCCAAACCAGAAGGCCGGTTTCAAGGCATGGTCATTGGTATCATGAGTGCCCTAGTGGGTATCCCTG
TATTGCTGCTGCTGGCCTGGGCCCTAGCTGTCTTCTGCTCAACAAGTATGTCAGAGGCCAGAGGAGCT
GGAAGCAAGGACGACACTCTGAAGGAGGAGCCTTCAGCAGCACCTGTCCCTAGTGTGGCCTATGAGG
AGCTGGACTTCCAGGGACGAGAGAAGACACCAGAGCTCCCTACCGCCTGTGTGCACACAGAATATGC
CACCATTGTCTTCACTGAAGGGCTGGGTGCCTCGGCCATGGGACGTAGGGGCTCAGCTGATGGCCTGC
AGGGTCCTCGGCCTCCAAGACATGAGGATGGACATTGTTCTTGGCCTCTT

mGFP sequence:
ATGAGCGGGGGCGAGGAGCTGTTCGCCGGCATCGTGCCCGTGCTGATCGAGCTGGACGGCGACGTGC
ACGGCCACAAGTTCAGCGTGCGCGGCGAGGGCGAGGGCGACGCCGACTACGGCAAGCTGGAGATCAA
GTTCATCTGCACCACCGGCAAGCTGCCCGTGCCCTGGCCCACCCTGGTGACCACCCTCTGCTACGGCAT
CCAGTGCTTCGCCCGCTACCCCGAGCACATGAAGATGAACGACTTCTTCAAGAGCGCCATGCCCGAGG
GCTACATCCAGGAGCGCACCATCCAGTTCCAGGACGACGGCAAGTACAAGACCCGCGGCGAGGTGAA
GTTCGAGGGCGACACCCTGGTGAACCGCATCGAGCTGAAGGGCAAGGACTTCAAGGAGGACGGCAAC
ATCCTGGGCCACAAGCTGGAGTACAGCTTCAACAGCCACAACGTGTACATCCGCCCCGACAAGGCCAA
CAACGGCCTGGAGGCTAACTTCAAGACCCGCCACAACATCGAGGGCGGCGGCGTGCAGCTGGCCGAC
CACTACCAGACCAACGTGCCCCTGGGCGACGGCCCCGTGCTGATCCCCATCAACCACTACCTGAGCAC
TCAGACCAAGATCAGCAAGGACCGCAACGAGGCCCGCGACCACATGGTGCTCCTGGAGTCCTTCAGC
GCCTGCTGCCACACCCACGGCATGGACGAGCTGTACAGGTCCGGACTCAGA

Then, a lentivirus was constructed by applying the lentivirus vector encoding PD-1–GFP. After treating the virus on NK-92 cells, polybrene (Millipore) was added to a final concentration of 8 μg/ml and spinoculation was performed at 360×g for 90 min at 32 °C to increase infection efficiency. Infected NK-92 cells were sorted for the overexpression of GFP using MoFlo Astrios EQ (Beckman Coulter) and cultured in NK-92 complete culture medium maintained with 1 μg/ml puromycin. EVs were collected in the same way as described for NK-EVs without PD-1.

Preparation of synthetic liposomes

For the preparation of liposomes, 2 mg of 1-palmitoyl-2-oleoyl-sn-glycero-3-phosphocholine (POPC) (Avanti, 850457C) was dissolved in chloroform and completely dried by evaporation under vacuum. The lipid film was hydrated with 1 ml of PBS and disrupted by sonication, and the resulting suspension was extruded through a filter with 100-nm pores (Avanti, 610005) using a mini-extruder (Avanti, 610023).

Nanoparticle tracking analysis (NTA)

For NTA measurements, vesicle samples were diluted with filtered PBS to ~10^9 particles/ml and illuminated with 405-nm laser in the NTA equipment (Nanosight, LM10-HS). About 30 measurements were performed to analyze each vesicle sample.

Labeling with PKH67 using a standard protocol

For labeling with PKH67 following a standard protocol ("Protocol Guide: Exosome Labeling Using PKH Lipophilic Membrane Dyes" on sigmaaldrich.com), NK-CDVs were first pelleted by centrifugation at 18,000×g for 80 min. The pellets were resuspended with 100 μl of Diluent C (Sigma, CGLDIL), mixed with 0.6 μl of PKH67 dye (Sigma, MIDI67) by gentle pipetting, and

incubated for 5 min at room temperature. The reaction was quenched by adding 200 µl of 10% bovine serum albumin (BSA) (Biosesang, A1025) in PBS, and the total volume of the sample was increased up to 1 ml by adding Dulbecco's Modified Eagle's Medium (DMEM) (Sigma, D6429). The labeled CDVs were collected and excess dyes were cleared by centrifugation at 18,000×g for 2 h at 4 °C. The pellets were resuspended in 900 µl of PBS and 75 µl of DMEM. The sample was purified and concentrated again using an Amicon filter (10-kDa cutoff, Millipore, UFC5010) at 3,000×g for 40 min. After purification, the labeled CDVs were diluted to 100 µl with PBS. A sample with CDV-free media was prepared by the same procedure for the negative control.

TIRF microscopy and image analysis

Sample slides for TIRF imaging were prepared as previously described.[4] Briefly, flow cells were assembled from a glass coverslip and a glass slide bonded together using double-sided tape. Glass surface was coated with polyethylene glycols (PEG) (Laysan Bio, mPEG-SVA, MW 5,000) to avoid excessive nonspecific adsorption of vesicles. For the surface attachment with antibody, 3% of biotinylated PEG (Laysan Bio, BIO-PEG-SVA, MW 5,000) was included to tether NeutrAvidin (Thermo Fisher, 31000). For the imaging of free-floating vesicles, vesicles were observed immediately after injection. For the imaging of surface-bound vesicles, DiI-labeled vesicles were tethered on PEG-coated glass surface by using a biotinylated CD63 antibody (BioLegend, 353017) and imaged. For PD-1 detection experiments (Figure 5), PerCP-Cy5.5-conjugated anti-PD-1 (BioLegend, 135208) was additionally introduced, incubated for 10 min, and washed. The slides were imaged with a custom TIRF microscope (Olympus IX73) equipped with a 60× oil-immersion lens (Olympus) for both illumination and observation. Samples were illuminated by the 488-, 532- and 633-nm CW lasers (Cobolt) and the resulting fluorescent images were acquired by an sCMOS

camera (Teledyne Photometrics, Prime BSI Express) typically with 100-ms resolution and ~100-μm field of view. For quantification of fluorescence, initial 10 frames of movies were averaged and either the number of fluorescent spots or the total intensity over the entire area was measured using custom MATLAB codes.

Estimation of fluorescent labeling efficiency

For photobleaching experiments, the labeled vesicles captured by anti-CD63 were continuously illuminated and the resulting changes in fluorescent intensities from individual spots were tracked. The signal from individual DiI molecules were measured both from the step size during photobleaching events and from the distribution of intensities that showed equally spaced Gaussian distributions. The results from two approaches agreed well and yielded ~65 as the single-dye fluorescence. Finally, the initial intensity from all vesicles were collected (mean ~ 122) and the labeling efficiency was estimated as 122/65 = 1.9. Additionally, the intensity distribution discretized by the step size was well fitted with a Poisson distribution with a mean of 1.9 (Figure S2E). A single phospholipid molecule is expected to occupy 0.629 nm^2 in lipid vesicles,[5] and the surface area of a small, 100-nm vesicle is $4\pi\times((100\ \text{nm})/2)^2 \sim 3\times10^4\ \text{nm}^2$. Therefore, we estimate that each DiI-labeled vesicle contains $\sim10^5$ molecules of lipid in the two leaflets. As a result, the mole fraction of DiI in vesicles (less than 10 molecules per vesicle) in DiI-CDV would be lower than 10^{-4}.

Supplementary Figures

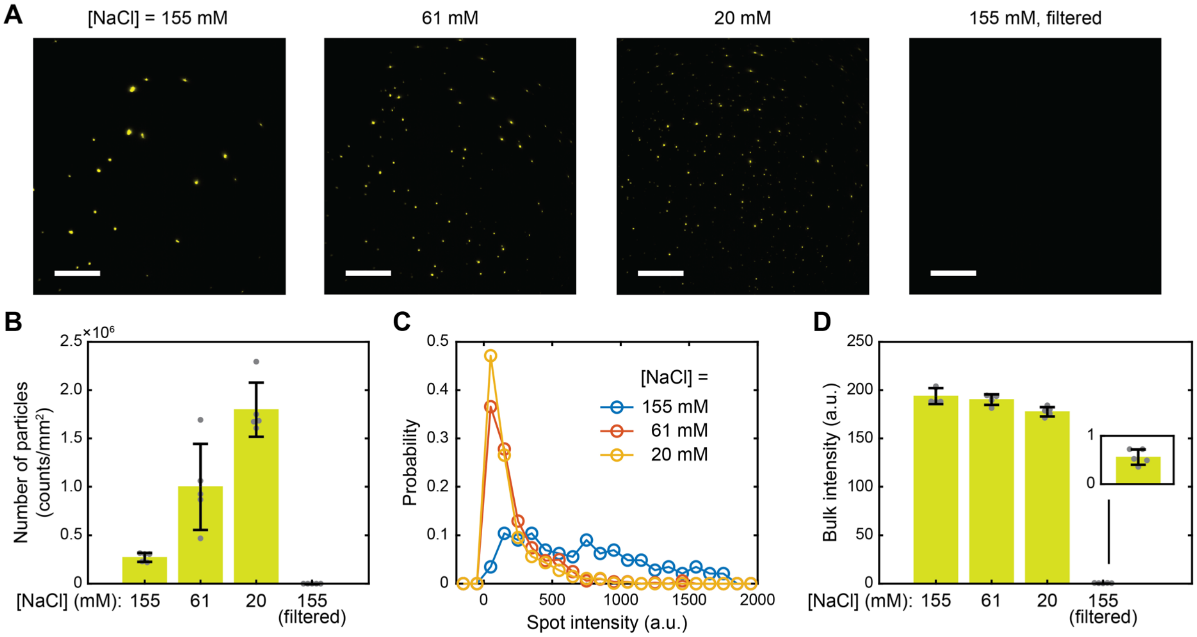

Figure S1. NaCl dependence of DiI aggregation

(A) Representative fluorescent images of 2 μM DiI observed at the indicated concentrations of NaCl. Scale, 20 μm. (B) The number of DiI particles with varying concentrations of NaCl. Error bars, mean ± s.d. of n = 5 measurements. (C) Distribution of fluorescent intensity of the DiI particles observed in (B). n = 144, 525, and 942 particles for 155, 61, and 20 mM NaCl, respectively. (D) The bulk fluorescence intensity of DiI solution after complete solubilization in detergent with indicated NaCl concentration. Inset shows a magnified view of the filtered 155 mM bar, which indicates that the remaining DiI concentration was ~5 nM. Error bars, mean ± s.d. of n = 5 images.

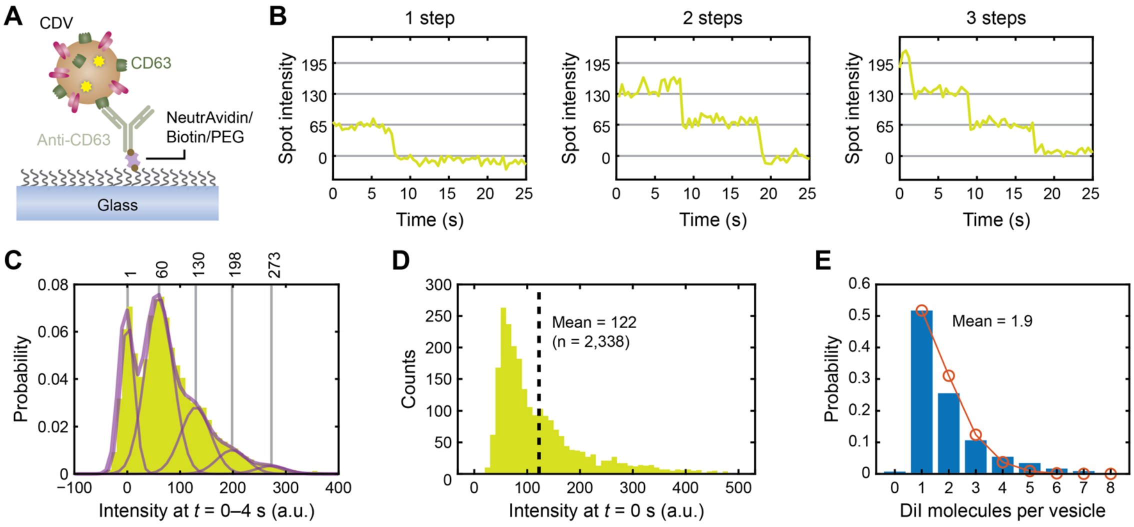

Figure S2. Estimation of labeling efficiency

(A) Schematic of vesicle capture for the measurement of labeling efficiency. DiI-labeled NK-CDVs were tethered by an antibody to the surface protein CD63. (B) Representative fluorescence intensity traces of single DiI-labeled vesicles with the indicated numbers of photobleaching steps. The excitation laser power was maintained the same to keep the rate of photobleaching constant, but photobleaching events are stochastic in nature and the waiting time to photobleaching was random. (C) Distribution of single-vesicle fluorescence in the early frames (0–4 s) of imaging. The distribution was fitted with a 5-component Gaussian mixture model (*purple*) and the locations of peaks were used to calculate the photobleaching step size. (D) Distribution of single-vesicle fluorescence at the beginning of imaging ($t = 0$ s). (E) Distribution of the estimated number of dye molecules. The intensity distribution in (D) was discretized by normalizing it to the photobleaching step size (65) measured in (B) and (C). Red curve indicates a Poisson distribution with a mean of 1.9.

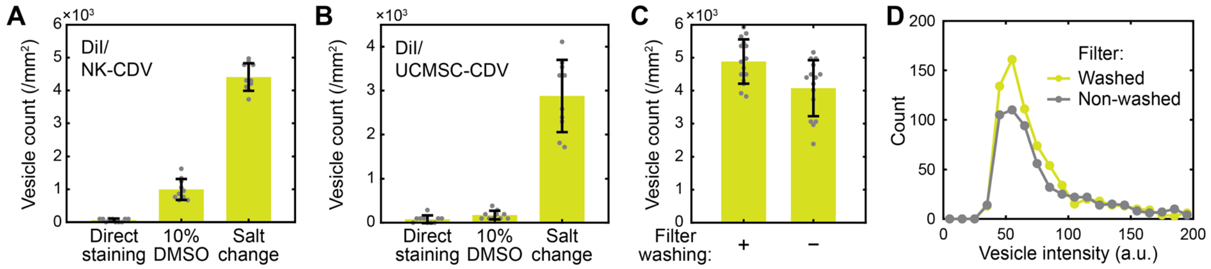

Figure S3. Comparison of CDV labeling efficiency

(A, B) Labeling efficiencies obtained with direct staining, the use of 10% DMSO, and salt-change method were compared for NK-CDVs (A) and UCMSC-CDVs (B). For the DMSO method, 10% DMSO was added to the staining buffer to improve the solubility of DiI. Error bars, mean ± s.d. of n = 10 images. (C, D) Effect of syringe filter washing on labeling results. For the washed case, cellulose acetate filters for dye removal (Advantec, 03CP020AS) were pre-washed with 2 ml of PBS and used for salt-change labeling.

Supplementary Video Legends

Video S1. Comparison of NK-CDVs labeled by direct staining and salt-change method

DiI-labeled NK-CDVs prepared by conventional direct staining method (*left*) and by salt-change method (*right*) are shown. Images were recorded with a TIRF microscope at 30-ms resolution. Scale, 10 μm.

Video S2. Effect of DiI concentration on salt-change labeling of NK-EVs

NK-cell-derived EVs were labeled by salt-change method with DiI prepared at 2 μM (*left*), 0.67 μM (*center*), and 0.2 μM (*right*) and imaged on a TIRF microscope at 10-ms resolution. Scale, 20 μm.

Supplementary References

(1) Jang, S. C.; Kim, O. Y.; Yoon, C. M.; Choi, D.-S.; Roh, T.-Y.; Park, J.; Nilsson, J.; Lötvall, J.; Kim, Y.-K.; Gho, Y. S. Bioinspired Exosome-Mimetic Nanovesicles for Targeted Delivery of Chemotherapeutics to Malignant Tumors. *ACS Nano* **2013**, *7* (9), 7698–7710. https://doi.org/10.1021/nn402232g.

(2) Choi, D.; Go, G.; Kim, D.-K.; Lee, J.; Park, S.-M.; Di Vizio, D.; Gho, Y. S. Quantitative Proteomic Analysis of Trypsin-Treated Extracellular Vesicles to Identify the Real-Vesicular Proteins. *J. Extracell. Vesicles* **2020**, *9* (1), 1757209. https://doi.org/10.1080/20013078.2020.1757209.

(3) Kim, O. Y.; Park, H. T.; Dinh, N. T. H.; Choi, S. J.; Lee, J.; Kim, J. H.; Lee, S.-W.; Gho, Y. S. Bacterial Outer Membrane Vesicles Suppress Tumor by Interferon-γ-Mediated Antitumor Response. *Nat. Commun.* **2017**, *8* (1), 626. https://doi.org/10.1038/s41467-017-00729-8.

(4) Lee, H.-W.; Choi, B.; Kang, H. N.; Kim, H.; Min, A.; Cha, M.; Ryu, J. Y.; Park, S.; Sohn, J.; Shin, K.; Yun, M. R.; Han, J. Y.; Shon, M. J.; Jeong, C.; Chung, J.; Lee, S.-H.; Im, S.-A.; Cho, B. C.; Yoon, T.-Y. Profiling of Protein–Protein Interactions via Single-Molecule Techniques Predicts the Dependence of Cancers on Growth-Factor Receptors. *Nat. Biomed. Eng.* **2018**, *2* (4), 239–253. https://doi.org/10.1038/s41551-018-0212-3.

(5) Nagle, J. F.; Zhang, R.; Tristram-Nagle, S.; Sun, W.; Petrache, H. I.; Suter, R. M. X-Ray Structure Determination of Fully Hydrated L Alpha Phase Dipalmitoylphosphatidylcholine Bilayers. *Biophys. J.* **1996**, *70* (3), 1419–1431. https://doi.org/10.1016/S0006-3495(96)79701-1.
